# Unveiling Fall Triggers in Older Adults: A Machine Learning Graphical Model Analysis

**DOI:** 10.3390/math12091271

**Published:** 2024-04-23

**Authors:** Tho Nguyen, Ladda Thiamwong, Qian Lou, Rui Xie

**Affiliations:** 1 Department of Statistics and Data Science, University of Central Florida, Orlando, FL 32816, USA; 2 College of Nursing, University of Central Florida, Orlando, FL 32816, USA; 3 Department of Computer Science, University of Central Florida, Orlando, FL 32816, USA

**Keywords:** undirected graphical models, mixed graphical models, machine learning, correlation analysis, fall risks, older adults, aging research, 62-08

## Abstract

While existing research has identified diverse fall risk factors in adults aged 60 and older across various areas, comprehensively examining the interrelationships between all factors can enhance our knowledge of complex mechanisms and ultimately prevent falls. This study employs a novel approach—a *mixed undirected graphical model* (MUGM)—to unravel the interplay between sociodemographics, mental well-being, body composition, self-assessed and performance-based fall risk assessments, and physical activity patterns. Using a parameterized joint probability density, MUGMs specify the higher-order dependence structure and reveals the underlying graphical structure of heterogeneous variables. The MUGM consisting of mixed types of variables (continuous and categorical) has versatile applications that provide innovative and practical insights, as it is equipped to transcend the limitations of traditional correlation analysis and uncover sophisticated interactions within a high-dimensional data set. Our study included 120 elders from central Florida whose 37 fall risk factors were analyzed using an MUGM. Among the identified features, 34 exhibited pairwise relationships, while COVID-19-related factors and housing composition remained conditionally independent from all others. The results from our study serve as a foundational exploration, and future research investigating the longitudinal aspects of these features plays a pivotal role in enhancing our knowledge of the dynamics contributing to fall prevention in this population.

## 1. Introduction

The likelihood of individuals encountering various health conditions increases significantly when aging, with some potentially occurring at the same time resulting from the cumulative impact of damage over time. Declining physical and mental abilities increase susceptibility to diseases, and ultimately, mortality is inevitable. Common health issues in the elderly such as frailty and falls are the consequences of multiple underlying factors; in fact, falls among elderly individuals have been a serious public health concern, with millions of occurrences each year [1]. Although not all falls are severe, most of them require medical treatment and activity restrictions due to the age of this population. Many incidents lead to fatal or nonfatal injuries, the restriction of mobility, and a reduction in the quality of life, as well as medical costs totaling billions of dollars in the United States of America. Numerous impairments, disabilities, and medical conditions have been known to be frequently related to the risk of falls in older adults [1]. Life factors such as living environment, social and/or psychological status, and technological developments have long been studied to show the associations between falling and fall-related problems [2,3]. Body composition has been linked to chronic diseases and mortality in the elderly; it is also a frailty biomarker and risk factor for falls [4]. Typical measurements such as fat mass and body mass index were correlated with balance impairment [5]. Furthermore, the recent global pandemic of SARS-CoV-2 (COVID-19) has tremendously impacted this population [6]. A report from the Center for Disease Control and Prevention (CDC) in March 2020 also revealed that individuals aged 65 and above accounted for 31% of COVID-19 infections, 45% of hospitalizations, 53% of intensive care unit admissions, and 80% of deaths related to this infection in the United States [7]. Advanced age and immune-compromised conditions, especially in those with chronic health conditions, are associated with the severity and fatality of COVID-19, making older adults a vulnerable population and susceptible to the infection [8,9]. The social and physical distancing recommendations from the CDC have limited their physical activity level which, as a result, is likely to affect their physical functioning and lead to a higher risk of falling [10,11]. Either considering intrinsic or extrinsic factors, it is important to recognize that the prevention of falls should target all etiologies of postural instability. This is especially vital for aging research and body composition analysis, which have established connections to various diseases, including cardiovascular diseases and diabetes, two other common chronic diseases among elders [12,13]. Identifying a broad spectrum of factors that can influence fall occurrences would improve the assessment of the possibility of occurrence, which would be valuable in minimizing incidents and guiding prevention plans.

### 1.1. Related Studies

The use of machine learning (ML) has been significantly developed and widely applied in medicine [14,15] and public health research over the past few years, particularly in aging research. Chen et al. (2023) delved into interpretable ML for fall prediction among older adults in China, aiming to create a model capable of accurately predicting falls and fall-related injuries using various ML algorithms while also providing understandable insights into predictive factors [16]. Similarly, Savadkoohi et al. (2021) employed deep neural networks to forecast human fall risk based on force-plate time series signals, showcasing the promising potential for accurately predicting fall risk through advanced ML techniques [17]. Likewise, the authors of Speiser et al. (2021) discussed ML’s application in developing prediction models for serious fall injuries, emphasizing the importance of leveraging sophisticated computational methods to identify and forecast fall risks among aging populations [18]. Odden and Melzer (2019) underscored ML’s growing importance, particularly in facilitating personalized interventions within aging-related studies [19]. While existing research primarily focuses on supervised ML, there is a gap in studies utilizing unsupervised ML to address a wide range of fall risk factors. Das and Dhillon (2023) revealed that half of the reviewed studies used supervised ML, among which logistic regression, random forest, and XG Boost are frequently used methods [20]. Moreover, the existing literature tends to evaluate a restricted number of variables. There is a possibility that these variables may interact with each other, contributing to fall occurrences. This presents a notable research gap in the field, as it limits the comprehensive exploration of potential predictors and their interactions in relation to fall risk among older adults. Hence, there is a necessity to construct a data analysis model that comprehensively grasps the associations among pertinent variables associated with falls. Incorporating unsupervised ML techniques and adopting a broader scope of risk factors could offer novel insights into comprehensive fall risk assessments and further enhance fall prevention strategies for older adults.

### 1.2. Study Objectives

As modern healthcare generates an enormous volume of data, employing appropriate analytical methods is crucial to extract the maximum insights from the collected information. ML accomplishes the following: ① ML addresses the challenges in high-dimensional statistics, in which the number of experimental units or observations in the data is significantly smaller than the number of measurement variables/features; ② ML is equipped to investigate data sets containing various types of variables/features including continuous, categorical, and count variables; ③ ML easily gains a comprehensive understanding of complicated interactions; ④ ML unravels nonlinear and higher-order relationships, of which traditional correlation analysis is struggling to solve; and ⑤ 5 ML is flexible from a priori assumptions such as the type of distribution, the additivity of the parameters, the linearity of predictors in regression, or homoscedasticity [21]. Consequently, ML has the ability to handle numerous variables and complex interactions, opening doors to discovering unexpected relationships between factors and avoiding the limitations of pre-selecting predictors based on prior hypotheses.

In our study, we proposed the utility of a novel unsupervised ML method, namely *mixed undirected graphical models*, to analyze structured data and find patterns in the features of fall risk prevention studies [22]. This approach highlights the enhanced effectiveness of ML compared to traditional correlation techniques, as it is adaptable and can work with various types of data distributions. The graphical model not only capitalizes on the strengths of ML but also offers improved and innovative methods for illustrating meaningful relationships between various factors contributing to falls. Additionally, the graphical model is well-suited for handling a vast number of domains, which allows a thorough investigation of numerous risk factors simultaneously. To the best of our knowledge, this study represents one of the first attempts to explore fall risk factors in this context. The findings of this study provide meaningful insights into the application of ML in understanding the complicated structure and patterns of variables. They also serve as stepping stones for future research, such as exploring causal or temporal relationships, by highlighting important risk factors and assisting in the design of interventions aimed at effectively reducing the occurrence of falls among older adults.

## 2. Materials and Proposed Method

### 2.1. Study Design and Participants

A total of 121 community-dwelling individuals aged 60 and older were enrolled from the central region of Florida, USA, starting in 2021 and ending in 2023. The recruitment criteria include meeting the low-income criteria based on the 2019 United States Census guidelines, being capable of walking independently without assistance from another person, having no cognitive impairment, residing independently, and being fluent in English and/or Spanish. Participants were asked to complete self-report questionnaires of sociodemographic characteristics, self-rated health, the Fatigue, Resistance, Ambulation, Illnesses, and Loss of weight scale (FRAIL), the Short Falls Efficacy Scale International (short FES-I), COVID-19-related questions, the Patient Health Questionnaire (PHQ-9), the Geriatric Anxiety Inventory Short Form (GAI-SF), the Mindful Attention Awareness Scale (MAAS), the CDC Stopping Elderly Accidents, Deaths, and Injuries (STEADI), and the Rapid Assessment of Physical Activity (RAPA), followed by the assessment of grip strength, the Balance Tracking Systems (BTrackS) test, and the 30 s Sit-To-Stand (STS) test. Details on each questionnaire and physical assessment are described in the following section. In addition, each individual was provided with a wrist-worn accelerometer and given instructions on how to wear it for 7 days [23]. The body composition was recorded using a portable bioelectrical impedance analysis device, and participants were instructed per the manufacturer’s guidelines [5,24].

### 2.2. Study Variables/Features

The data set consists of 135 features. These measurements were drawn from diverse disciplines, i.e., self-report questionnaires regarding sociodemographic and general health, psychological status, COVID-19-related questions, fall risk self-assessments, body composition measurements, body balance performance tests via the BTrackS^™^ Assess Balance System, and accelerometer-based physical activity levels [25]. These disciplines encompassed various domains and different distribution types including continuous, categorical, and count data.

- ***Sociodemographic and Self-rated Health***. Sociodemographic variables were obtained using a self-report questionnaire [22]. Participant age was collected numerically and gender categorically. Participant’s race consisted of White and Non-White, where the Non-White category included Hispanic, African-American, and Asian older adults. The level of education was divided into two categories, high school or below and college or higher. Financial difficulty was categorized as adequate or less and more than adequate. Household composition was defined as living alone or living with others. Self-rated health status was acquired using a five-point Likert scale, and participants were classified as excellent, very good, or good or below.
- ***Psychological Status***. Depressive symptoms were assessed using the self-report PHQ-9, which consists of nine items assessed using a four-point scale [26,27]. Anxiety was assessed by the GAI-SF, which comprises only five of the original items, has a closed-choice response format (yes/no), and is scored in a single direction [28]. Additionally, participants’ attention and awareness of present occurrence (mindfulness) were evaluated using the MAAS, a fifteen-item questionnaire on a six-point scale [29]. These features are continuously distributed.
- ***Body Composition Measurements***. Height was measured in centimeters and weight was measured in kilograms with no shoes. The body mass index was calculated as the weight divided by the square of height (kg/m^2^). The body composition measurements comprised whole-body, trunk, and both sides’ limbs at six different frequencies (1, 5, 50, 250, 500, and 1000 kHz). Fat and water measurements were recorded including intracellular water, extracellular water, total body water, body fat mass, lean body mass, dry lean mass, skeletal muscle mass, skeletal muscle index, visceral fat level, visceral fat area, and basal metabolic rate results.
- Fall Risks’ Self-assessments and Performance Tests.
  - Frailty was evaluated through the FRAIL scale, a self-report questionnaire comprising five items assessing fatigue, resistance, ambulation, illnesses, and weight loss [30].
  - The short FES-I questionnaire was employed to assess the fear of falling, consisting of seven items measuring the level of concern related to falling during the performance of daily activities on a four-point Likert scale [31].
  - The STEADI algorithm is a self-risk checklist consisting of twelve questions that focus on fall risk factors [32].
  - The brief version of the Senior Technology Acceptance (STA) was employed to measure older adults’ acceptance of technology. The questionnaire contains four domains with fourteen items on a ten-point scale [33].
  - For performance tests, grip strength, an indicator of hand and forearm muscle strength, was collected numerically on both sides using a hydraulic hand dynamometer [34]. The 30 s STS test (also called the chair-stand test) was used to assess dynamic balance. Participants were directed to cross their arms over their chest, stand away from a chair, and return to a sitting position as many times as possible within 30 s. Any use of hands during the test resulted in a score of zero [35]. The BTrackS balance assessment consists of four 20 s trials, measuring postural sway by tracking the center of pressure on a force platform. The first trial is for familiarity, and each trial requires the participants to stand as still as possible on the balance plate with hands on their hips, eyes closed, and feet shoulder width apart [36].
- ***COVID-19 related questions***. Participants were asked whether they had ever tested positive for COVID-19 and to rate their perception of COVID-19 severity in their community over the past month on a four-point Likert scale. Fear of COVID-19 was evaluated using the Fear of COVID-19 Scale (FCV-19S), a seven-item, four-point Likert scale adapted from [37].
- ***Accelerometer Data and Physical Activity Level***. The processing of accelerometer data was carried out using the R package *GGIR* (version 2.4-0) [38], in which the minutes (per week) spent in sedentary behavior (SB), light physical activity (LPA), and moderate-to-vigorous physical activity (MVPA) were recorded. RAPA, a nine-item, self-administered questionnaire, was utilized to evaluate a wide range of physical activity levels, from sedentary to vigorous activity (the first seven questions; the total score is out of seven), as well as strength and flexibility training (scored separately; strength training = 1, flexibility = 2, both = 3) [39].

### 2.3. Graphical Models

**Figure 1.**
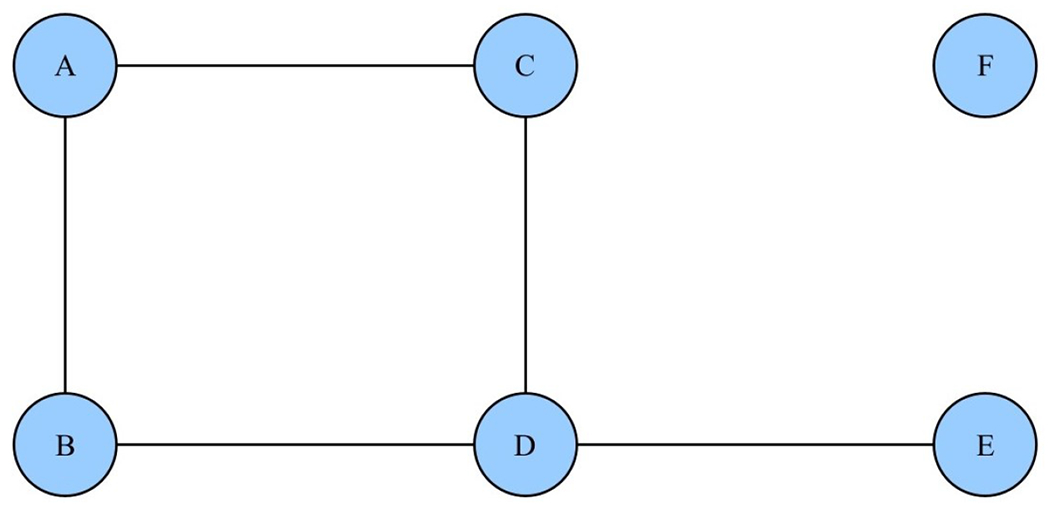
An example of an undirected graphical model. There are multiple ways to express the conditional dependencies/independencies among nodes. A and B are adjacent, denoted *A*~*B*. Nodes C and E are conditionally independent given D, denoted C⊥E∣D. Node F is independent of each of the other nodes.

The *mixed undirected graphical model* (MUGM) was utilized to analyze the associations between fall risk factors. The graphical model is an unsupervised machine learning method capable of revealing the joint probability distribution and the strength, as well as the signs of the relationships among the entire set of random variables [40]. In undirected graphical models (also known as Markov random fields or Markov networks), each node represents a random variable, and the undirected edges represent association between variables. Two nodes are adjacent if there is an edge joining them, denoted X∼Y. Consequently, the absence of an edge implies conditional independence, given the other variables, expressed as X⊥Y|rest. A simple example of an undirected graphical model is illustrated in Figure 1.

The probability density function *f* over an undirected graph can be presented as follows:

(1) $$f(x)=\frac{1}{Z}\underset{C\in 𝒞}{\prod }\psi_{C}\left(x_{C}\right)$$

where 𝒞 is a collection of cliques of the graph, $\psi_{C}\left(x_{C}\right)$ is a non-negative potential function (or clique potential), and *Z* is a normalization constant (or partition function) obtained by integrating or summing the product with respect to *x*_*C*_ [40]. A graphical model can specify the higher-order dependence structure of a joint probability distribution [40]; however, only pairwise mixed undirected graphical models or, at most, second-order interactions are presented in this study. As a result, the complete subgraph in Figure 1 can be derived as follows:

(2) $$f(a,b,c,d)=\frac{1}{Z}\psi (a,b)\psi (a,c)\psi (a,d)\psi (b,c)\psi (b,d)\psi (c,d)$$

where ψ(⋅,⋅) is a non-negative potential function of two input nodes [40].

Furthermore, mixed graphical models are able to learn over a combination of continuous and discrete variables [40]. Lee and Hastie (2015) [41] parameterize a pairwise mixed graphical model on ρ continuous variables (*x*) and *q* categorical variables (*y*). The model is derived with density:

(3) $$p(x,y;\text{Θ})\propto \exp\left(\overset{p}{\underset{s=1}{\sum }}\overset{p}{\underset{t=1}{\sum }}-\frac{1}{2}\beta_{st}x_{s}x_{t}+\overset{p}{\underset{s=1}{\sum }}\alpha_{s}x_{s}+\overset{p}{\underset{s=1}{\sum }}\overset{q}{\underset{j=1}{\sum }}\rho_{sj}\left(y_{j}\right)x_{s}+\overset{q}{\underset{j=1}{\sum }}\overset{q}{\underset{r=1}{\sum }}\phi_{rj}\left(y_{r},y_{j}\right)\right)$$

where *x*_*s*_ denotes the *s*th of *p* continuous variables, and *y_j_* denotes the *j*th of *q* discrete variables. The joint model is parameterized by $\text{Θ}=\left[\left\{\beta_{st}\right\},\left\{\alpha_{s}\right\},\left\{\rho_{sj}\right\},\left\{\phi_{rj}\right\}\right]$, in which $\rho_{sj}\left(y_{j}\right)$ is a function taking *L_j_* values $\rho_{sj}(1),\ldots ,\rho_{sj}\left(L_{j}\right)$, and $\phi_{rj}\left(y_{r},y_{j}\right)$ is a bivariate function taking on $L_{r}\times L_{j}$ values, or, equivalently, $\rho_{sj}\left(y_{j}\right)$ is a vector of length *L_j_*, and $\phi_{rj}\left(y_{r},y_{j}\right)$ is a matrix of size $L_{r}\times L_{j}$ [41]. The discrete *y_r_* takes on *L_r_* states. The model parameters are $\beta_{\mathrm{st}}$ continuous–continuous edge potential, $\alpha_{s}$ continuous node potential, $\rho_{sj}\left(y_{j}\right)$ continuous–discrete edge potential, and $\phi_{rj}\left(y_{r},y_{j}\right)$ discrete–discrete edge potential.

The conditional distributions are given by linear regression and multiclass logistic regressions. The model acts as an extension of two well-known single-modal models to the multi-modal domain. It simplifies to a multivariate Gaussian distribution in the case of only continuous variables and to the Ising model when all the variables are discrete or categorical, which are a special case of log-linear models for multiway contingency tables [41,42].

### 2.4. Data Analysis Methods

#### 2.4.1. Exploratory Data Analysis

Descriptive statistics of participants’ sociodemographic, self-report questionnaires, and performance tests are presented as the median and interquartile range for continuous variables and as frequency and percentage for categorical variables. Missing data were imputed by the variable’s mean, as there was less than 5% of missingness in only one variable, which captured the number of falls that caused an injury during the previous year. Spearman’s rank correlation was performed to explore the relationships between continuous variables. The strength of the correlation coefficient was interpreted as weak (*ρ* ≤ 0.49), moderate (*ρ* = 0.50 – 0.70), or strong (*ρ* ≥ 0.70). All analyses were performed using R Statistical Software (version 4.2.0) [43], and a *p*-value < 0.05 was considered statistically significant.

#### 2.4.2. Exploratory Factor Analysis on Body Composition Variables

Exploratory factor analysis (EFA) was performed on the body composition variables using the functions fa() and fa.diagram() for visualization in the R package *psych* (version 1.7.8) [44]. EFA is an appropriate tool for assessing the significance of each dimension and examining the interconnections among dimensions. This method assumes several latent factors affecting the observed values and the study features. Extracted factors in EFA represent the dimensions they measure, effectively condensing data from multiple dimensions into a smaller set that accurately represents the broader variable group [45,46]. The factor model is presented by the following equation:

(4) $$y_{i}=\lambda_{i1}a_{1}+\lambda_{i2}a_{2}+\cdots +\lambda_{in}a_{n}+e_{i}$$

where *y* is an observed variable (indicator), *λ_ij_* is the loading of *i*th variable on the *j*th factor, *a* is a common factor, and *e* is the unique variance of *y* [45]. Variance can be partitioned into the following:

- Common variance is the amount of variance shared among a set of variables. Communality (or *h*^2^) is a common variance that ranges between 0 and 1, with closer to 1 suggesting that the extracted factors explain more of the variance of an individual item.
- Unique variance (or 1 − *h*^2^) consists of specific variance and error variance, and it is any portion of variance that is not common [46].

To determine the number of factors to retain, a scree plot was generated depicting eigenvalues that represent the amount of variation accounted for by each underlying factor. Subsequently, the Kaiser criterion (Kaiser–Guttman rule) was applied to enhance the determination, which ascertains the number of factors with eigenvalues greater than 1 [47,48].

#### 2.4.3. Model Development and Validation

Utilizing the data set consisting of extracted body composition factors from EFA, the remaining continuous and all categorical features were combined as inputs for running the undirected graphical model using the R package *mgm* (version 1.2-14) [49,50]. The MUGM is computed using *ℓ*_1_-regularization, which drives all parameter estimates toward zero and sets very small parameter estimates to exactly zero. The tuning parameter is *λ*, which controls the strength of the penalty. To ensure a sparse model, the negative log-pseudolikelihood is minimized with respect to *λ* [41]:

(5) $$\underset{\text{Θ}}{\min}\;ℒ(\text{Θ})+\lambda \left(\underset{t<s}{\sum }\left|\beta_{st}\right|+\underset{s,j}{\sum }\|\rho_{sj}{\|}_{2}+\underset{r<j}{\sum }\|\phi_{rj}{\|}_{F}\right)$$

where Θ is the parameter space for all of the model parameters. In [42], the authors modified the above algorithm so that it uses different sparsity penalties *λ* for the three edge types: edges connecting two continuous nodes $\left(\lambda_{cc}\right)$, edges connecting a continuous and discrete node (*λ_cd_*), and edges connecting two discrete nodes (*λ_dd_*).

We used ten-fold cross-validation [51] to select the optimal regularization parameters. This method involves dividing the data randomly into ten sets then estimating a graph based on the nine training sets and testing the negative log-likelihood on the remaining validation set. This process is repeated with each set serving as the testing fold, resulting in ten performance measurements. The optimal model parameter configuration was identified based on the best performance and subsequently utilized to retrain the model using the entire data set for final reporting. Other methods for choosing the regularization parameter include AIC (Akaike Information Criterion) [52], BIC (Bayesian Information Criterion) [53], StARS (Stability Approach to Regularization Selection) [54], and StEPS (Stable Edge-specific Penalty Selection) [42], which is a modification of the StARS approach. The latter two methods focus on selecting parameters to provide stable graphical estimations. The Extended Bayesian Information Criterion (EBIC) [55] was utilized for model selection, which performs better than the ordinary BIC in high-dimensional feature spaces.

The resulting graph was visualized using the R package *qgraph* (version 1.9.5) [56]. Variables in the same categories are positioned spatially close to each other. The thickness of the edges demonstrates the strength of partial association, i.e., the relationship between two variables while conditioning on all other variables. These edges are weighted; in other words, their strength is indicated by regression weights. Moreover, the colors of the lines indicate the magnitude of correlation, where green lines represent positive associations and red lines represent negative ones. The MUGM method is summarized in the Algorithm 1 below.

**Algorithm 1:** The Mixed Undirected Graphical Model (MUGM) Method

**INPUT**: The data set consists of 135 measurements from six categories with both continuous and categorical variables.
**Step 1**: Extract factors from the body composition measurements following Equation (4).
**Step 2**: Use the measurements from the remaining five categories and body composition factors to train the mixed undirected graphical model based on Equation (3) and Equation (5).
**Step 3**: Select the optimal regularization parameter *λ* in Equation (5) using ten-fold cross-validation.
**OUTPUT**: Corresponding undirected graph with nodes of the same category positioned closely.

## 3. Results

### 3.1. Participant Characteristics

One participant was excluded from the analysis due to missing not at random; hence, the final data set consisted of 120 (93 females and 27 males) older adults, whose mean age was 74.8 ± 7.38 and whose median age was 74 (IQR 69 – 79) years. Of the participants, 72.5% (87/120) were White, 71.7% (86/120) attended college or higher, 55% (66/120) had a more than adequate financial situation, 41.7% (50/120) lived alone, and 58.3% (70/120) were living with others. Notably, almost half of the participants (56/120, or 46.7%) rated their health as good or below. Among 120 participants, there were 16 (13.4%) who had two or more falls and 2 (1.6%) individuals who had two or more injurious falls during the past year. Furthermore, 45% (54/120) of participants were pre-frail or frail. Information on other self-assessment scores (i.e., psychological well-being status, fear of COVID, short FES-I, STEADI, STA, and RAPA), physical evaluation (i.e., grip strength, balance test, 30 s STS), and daily average physical activity levels processed from accelerometry devices are also presented in Table 1.

### 3.2. Mixed Undirected Graphical Models: Relationships between All Features

There was a total of 37 variables (9 categorical, 2 count, and 26 continuous) analyzed in the MUGM. Among the continuous variables, 7 were body composition factors resulting from the EFA (as described in the following sections). All study features were grouped, colored, and labeled into ① sociodemographic and self-rated health (light orange), ② psychological status (blue), ③ body composition measurement factors (green), ④ COVID-19-related questions (yellow), ⑤ fall risks’ self-assessments and performance tests (pink), and ⑥ accelerometer and physical activity level (orange).

**Figure 2.**
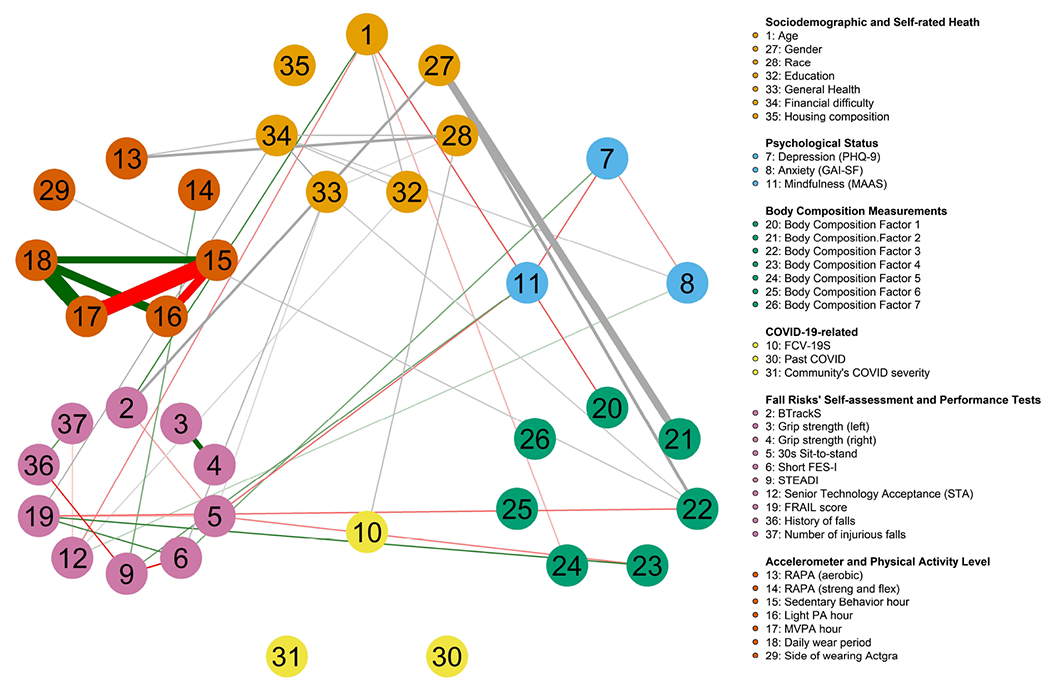
Mixed undirected graphical models estimated from 37 variables capturing various aspects including sociodemographic and self-rated health, psychological status, body composition measurement factors, COVID-19-related questions, fall risk self-assessments and performance tests, and accelerometer and physical activity level of 120 community-dwelling older adults.

Overall, 34 variables were identified as having pairwise relationships with one another (Figure 2). The proportion of non-zero edges was 9.934%, and the optimal regularization parameters *λ* with corresponding EBIC values were estimated nodewise. For example, the feature “Side of wearing Actgra” has *λ* = 0.016, with an EBIC value of −495.247. Prominent relationships include SB hour with significantly strong negative links to both LPA hour and MVPA hour, while the accelerometer’s wearing period showed strong positive links to all three physical activity level hours. Remarkably, the PHQ-9 score had pairwise negative correlations with all psychological scores but a positive correlation with the short FES-I score. Participant’s age was positively correlated to balance score but negatively correlated to body composition factor 1, body composition factor 5, and STA score. Notably, the FRAIL score was negatively associated with body composition factor 3 but positively associated with body composition factor 5 and the short FES-I score. The STEADI score had moderate positive relationships with the RAPA (strength and flexibility) and MAAS, while it had moderate negative relationships with the short FES-I score and history of falls. Past COVID infection, community COVID severity, and housing composition were the three nodes conditionally independent from all other variables.

The edges connecting categorical variables to continuous variables or other categorical variables are undefined, as they were computed from more than one parameter. Hence, a sign could not be assigned to these edges, as indicated with grey lines. Notably, among the intercorrelations, gender had a strong association with body composition factor 2, while having moderate associations with body composition factor 3 and BTrackS score. General health was connected to financial difficulty, 30 s STS, and short FES-I. Race and RAPA (aerobics), race and FCV-19S, FRAIL score and financial difficulty, and the side of wearing an accelerometer device and body composition factor 3 were other compelling moderate interrelationships.

### 3.3. Spearman’s Correlation and Correlation Matrix

**Figure 3.**
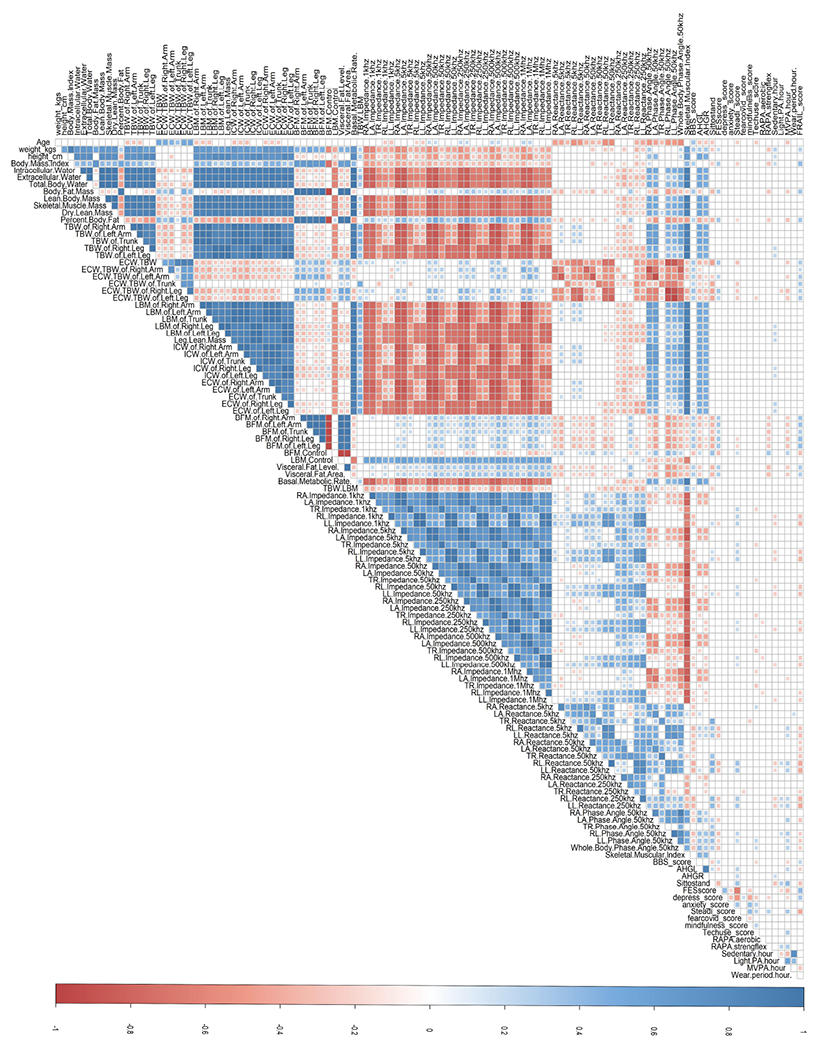
Spearman’s correlation coefficients matrix of 119 continuous variables. Only statistically significant correlations (*p* < 0.05) are illustrated. The intensity of color reflects the magnitude of the correlation, with red indicating negative relationships and blue indicating positive relationships.

Apparent patterns among body composition measurements were observed, indicating the potential presence of multicollinearity. A primary trend is observed where intracellular water, extracellular water, total body water, and lean body mass values of all segments present significant negative correlations with upper limbs’ impedance measurements across all frequencies (*ρ* < −0.7, *p* < 0.05). The ratio of extracellular water with total body water, body fat mass value, and visceral fat value, on the other hand, presented significant negative correlations with reactance measurements across all frequencies except in the 250 kHz frequency. Moreover, lean body mass, intracellular water, and extracellular water had significant positive correlations with skeletal muscle mass (*ρ* = 1.0, 1.0, 0.95, respectively, *p* < 0.05). Similarly, lean body mass, intracellular water, and extracellular water values had significant positive correlations with dry lean mass (*ρ* = 0.99, 0.99, 0.94, respectively, *p* < 0.05). Figure 3 demonstrates the results derived from Spearman’s correlation coefficients with only statistically significant correlations illustrated. The intensity of color reflects the magnitude of the correlation, with red indicating negative relationships and blue indicating positive relationships. The high multicollinearities observed among these body composition measurements suggested a necessity for variable reduction.

### 3.4. Exploratory Factor Analysis on Body Composition Measurements

**Figure 4.**
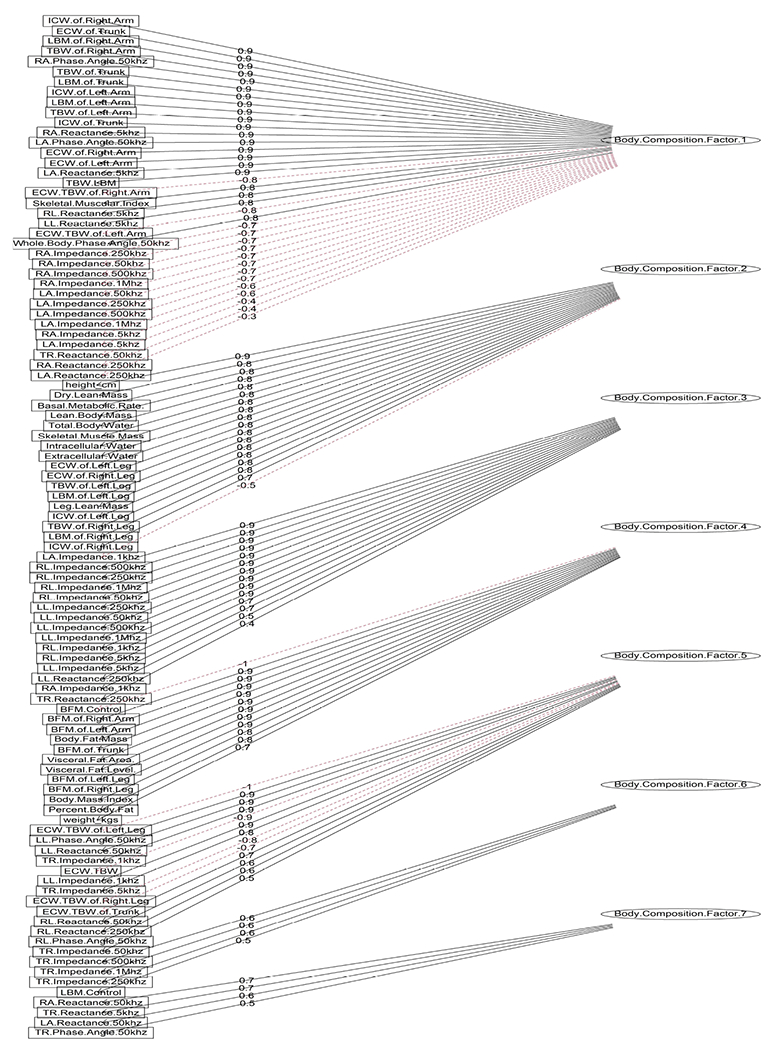
Diagram of factor loadings on observed variables. The strength of relationships ranges from −1 to 1, with black solid lines indicating positive relationships and red dotted lines indicating negative relationships.

In light of the correlation coefficients, the body composition variables were selected and their correlation matrix was used as the input to perform the exploratory factor analysis. The outcome from the Kaiser–Meyer–Olkin test (overall = 0.913) indicated a marvelous suitability of the subset, and Barlett’s test also indicated unequal variances across the sample (*p* < 0.001). To extract the common factors, the normal distribution of the observed variables was assumed to perform maximum likelihood, and a varimax was applied to orthogonally rotate the factors, ensuring equal correlation between them. Seven factors (whose eigenvalues > 1) were retained that are sufficient to explain the total amount of variance in the body composition subset. They cumulatively accounted for 89% of the variance in the subset; thus, the remaining factors account for a very small proportion of the variability (approximately 11%) and are potentially unimportant. The relationships between the factors and 101 observed body composition variables (also known as factor loadings) are illustrated in Figure 4, along with the strength which ranges from −1 to 1 and the magnitude in which red dotted lines indicate negative relationships and black solid lines indicate positive relationships. The seven factor scores were extracted and integrated into the original data set, replacing the body composition variables, and were named body composition factor 1 to body composition factor 7.

## 4. Discussion

### 4.1. Main Findings

Through a parameterized joint probability density, graphical models characterize the higher-order dependence structure and visually uncover the patterns of diverse data distributions. The ML-based approach is particularly useful when dealing with high-dimensional data sets and offers a wide range of applications that yield innovative and practical insights. With the vast amount of data generated in modern healthcare, it is essential to utilize suitable analytical methods to extract the most valuable data from the collected information. In this study, the mixed undirected graphical model was utilized to unravel the associations among various factors linked to the risk of falls in the elderly population. The study findings indicated interesting intercorrelations among these factors; for instance, psychological well-being, self-assessment fall risk, and body composition factors were, as expected, found to be connected to participants’ sociodemographic status, including age and gender, as well as how they rated their health and answered the self-assessment questionnaires. Particularly, the connection between participants’ frailty status, their body composition measurements, and financial status are other intriguing findings. The effect of psychological status on how individuals self-assessed their risk of falling indicated the intricate interplay between mental well-being and the perception of one’s susceptibility to falls. These findings shed light on the complex interactions between physical health, socioeconomic factors, and fall risks. On the contrary, COVID-19-related factors including past infection, perception of disease severity in the community in the past month, and housing composition are found to be independent of other variables. Since the questions are subjective and no environmental factors were investigated, it is important to examine the specific situations in which this independence holds. These features may not directly contribute to fall risk according to current data; however, their impact on health and well-being over time should not be overlooked. Future research could explore the potential pathways and mechanisms through which these factors may impact fall risk in the long term, which can offer valuable insights into comprehensive fall prevention strategies tailored to various contexts and demographic groups.

Understanding the strength and significance of these associations is crucial for developing effective intervention strategies to target specific risk factors, mitigate fall risks, and improve overall well-being and safety in this population. For example, one intervention could focus on balance-training exercises for older adults while also addressing nutrition and physical activity tailored to maintaining healthy body composition and stamina. Similarly, interventions targeting muscle strength, flexibility, and physical activity may benefit frail individuals. Given the associations between race and aerobic fitness (RAPA), COVID-19 stress (FCV-19S), financial difficulty, and body composition factors, interventions should adopt culturally sensitive approaches including community-based health programs, culturally tailored exercise and stress management interventions, and initiatives addressing socioeconomic disparities in health outcomes. Due to the complexity of the observed interrelationships, interventions would likely benefit from a multidisciplinary team approach involving healthcare providers, nutritionists, physical therapists, mental health professionals, and social workers. This collaborative approach ensures comprehensive assessment and personalized interventions that address individual needs across various health domains.

The application of graphical models reflects a growing trend in social sciences [57,58], and various health disciplines [14,15,59–61]. Particularly, Bhushan et al. utilized the Gaussian graphical model to explore and visualize the relationships between items and factors in environmental psychology research [57], and Kalisch et al. applied graphical models on the International Classification of Functioning, Disability, and Health data to visualize the dependence structure of the data set, dimension reduction, and comparison of subpopulations in studying human functioning [60]. In the field of aging research, specifically in assessing and preventing falls, numerous studies have employed conventional statistical methods [62,63], whereas the utilization of graphical models remains relatively uncommon. However, this approach has gained prominent attention and proves particularly beneficial when dealing with high-dimensional data sets, where conventional statistical methods often struggle to handle the complexity and multitude of variables in such data sets. Correlation coefficient tests and matrices can effectively uncover the linear relationships among continuous variables and offer an appealing representation of relationships. Nonetheless, their effectiveness diminishes when dealing with a large number of variables. Considering high-dimensional data, correlation matrices consisting of more than 100 pairs of factors become dense and impractical to illustrate the relationships. The massive volume of information can overwhelm the viewers and hinder the interpretation of nuanced patterns within the data. In the context of this data set, in which the number of features is much larger than the sample size, the interpretability of the correlation heatmaps was reduced and even posed additional challenges to interpret. In addition, the limitations of the statistical test resulted in the exclusion of discrete variables from the analysis, and none of the nonlinear relationships were analyzed. These limitations underscore the need for robust analytical techniques capable of handling the complexities inherent in high-dimensional data sets while ensuring a comprehensive exploration of all pertinent relationships. Addressing these gaps, the implementation of the undirected graphical models not only facilitated the efficient analysis of numerous and diverse features but also offered an intuitively understandable visual representation, which has captivated the interest of researchers. Furthermore, dimensionality reduction techniques such as EFA tackle the complexities in manifold data sets. EFA helps extract essential information and presents a condensed view of the relationships within the data. Accordingly, our proposed method tackles challenges that have remained unaddressed by previous studies and offers a comprehensive framework for understanding fall risk among older adults.

### 4.2. Limitations

Despite the novelty and advantages of applying the graphical model approach, it is crucial to acknowledge certain limitations. First, the Markov network performed in this study, while informative, does not establish a specific cause-and-effect relationship between variables, as it lacks directionality. Second, the observed number of connections between nodes in the MUGM graph is lower than anticipated, which raises important considerations about the structure and complexity of the relationships among variables. It could imply that the data exhibit more inherent sparsity or simplicity in relationships, which could have implications for model interpretation, generalization, and the underlying mechanisms driving the system being modeled. Therefore, it is a valuable insight that warrants further investigation and consideration in the analysis. Third, identifying and labeling the latent body composition factors requires a deeper understanding and additional knowledge to appropriately attribute meaning to the observed connections. Fourth, factors such as comorbidity and environmental exposures were not assessed, even though the literature recognized them as risk factors for falls [64,65]. Future endeavors could benefit from incorporating these factors to provide a more comprehensive understanding of fall risk among older adults. Lastly, the cross-sectional research design does not provide longitudinal information, and the pilot data set limited the diversity of the participant demographics, as the majority of the sample consisted of non-Hispanic White females. This demographic composition may limit the generalizability of the results to other gender and racial/ethnic groups of older adults.

## 5. Conclusions and Future Work

The utilization of machine learning, particularly graphical modeling, offers a promising avenue for studying complex relationships in high-dimensional data sets in the context of aging research. Employing an ML-based approach, we uncovered intricate associations among factors related to fall risks in the elderly. These included psychological well-being, the self-assessment of fall risk, and body composition, interconnected with age, gender, health perception, and financial status. However, COVID-19 factors and housing composition were found to be independent, highlighting the need for further exploration into their potential long-term impact on fall risks and the development of comprehensive prevention strategies. This study serves as a foundational exploration, with ongoing research playing a pivotal role in advancing the understanding of the dynamics contributing to the quality of life in older adults within a low-income community. Future study endeavors hold the potential to refine the exploration of factors related to fall risks by incorporating additional relevant nodes into the model, thereby enhancing its robustness, validity, and comprehensiveness. Moreover, understanding how these factors interact dynamically over time is crucial for developing targeted interventions and improving overall fall prevention strategies. Continuous efforts are dedicated to exploring the longitudinal aspects of these features through an ongoing clustered randomized controlled study design which aims to examine the effects of the technology-based intervention on fall risk among a more diverse and larger sample pool [66]. The approach delves into the temporal dynamics of dependencies among risk factors influencing fall prevention in this specific population. An optimal strategy involves integrating traditional statistical methods with machine learning techniques, ensuring that they offer added insights and contribute significantly to improving medical care outcomes. This integration is pivotal for leveraging the strengths of both methodologies and addressing the multifaceted challenges associated with fall prevention in older adults from low-income communities.

## Figures and Tables

**Table 1.** Descriptive statistics of participant characteristics in the 6 categories (*n* = 120).

Features	Participants, *n* = 120
**Sociodemographic**	
Age (Years)	
Mean (SD)	74.8 (7.38)
Median (IQR)	74 (69–79)
Gender	
Female	93 (77.5%)
Male	27 (22.5%)
Race/Ethnicity	
Non-Hispanic White	87 (72.5%)
Hispanic	21 (17.5%)
Others	12 (10%)
Education	
High school or below	34 (28.3%)
College or higher	86 (71.7%)
Financial difficulty	
Adequate or less	54 (45%)
More than adequate	66 (55%)
Living status	
Alone	50 (41.7%)
With others	70 (58.3%)
General health	
Excellent or very good	64 (53.3%)
Good or below	56 (46.7%)
**Psychological status**	
Depression PHQ-9 ^1^, median (IQR)	10 (9–12)
Anxiety GAI-SF ^2^, median (IQR)	10 (8.8–10)
Mindfulness MAAS ^3^, median (IQR)	81 (69.8–86)
**COVID-19-related**	
Fear of COVID-19, median (IQR)	14 (10–17)
**Self-assessment Fall risks**	
History of falls	
None	85 (70.8%)
One	19 (15.8%)
Two or more	16 (13.4%)
Number of injurious falls	
None	109 (90.8%)
One	9 (7.5%)
Two or more	2 (1.6%)
FRAIL ^4^	
Healthy	66 (55%)
Pre-frail or Frail	54 (45%)
Short FES-I ^5^, median (IQR)	9 (7–12)
STEADI^6^, median (IQR)	22.5 (21–24)
STA ^7^, median (IQR)	101 (90.8–112.3)
**Performance-based Fall risks**	
RAPA ^8^ Aerobics, median (IQR)	3 (2–3.3)
RAPA Strength and flexibility, median (IQR)	2.5 (0–3)
30 s sit-to-stand, median (IQR)	14.5 (12–17)
BTrackS ^9^ balance test, median (IQR)	27 (20–36)
Grip strength, left (kgs)	19.1 (15.8–24.9)
Grip strength, right (kgs)	20.6 (16.7–26.3)
**Accelerometer data**	
SB ^10^ (mins/day)	12.3 (11–13.6)
LPA ^11^ (mins/day)	3.4 (2.8–4.1)
MVPA ^12^ (mins/day)	0.7 (0.4–1)

1 PHQ-9, Patient Health Questionnaire.

2 GAI-SF, Geriatric Anxiety Inventory Short Form.

3 MAAS, Mindful Attention Awareness Scale.

4 FRAIL, Fatigue, Resistance, Ambulation, Illnesses, and Loss of weight scale.

5 FES-I, Falls Efficacy Scale International.

6 STEADI, Stopping Elderly Accidents, Deaths and Injuries.

7 STA, Senior Technology Acceptance.

8 RAPA, Rapid Assessment of Physical Activity.

9 BTrackS, Balance Tracking Systems.

10 SB, sedentary behavior.

11 LPA, light-intensity physical activity.

12 MVPA, moderate-to-vigorous-intensity physical activity.

## Data Availability

The data presented in this study are available on request from the corresponding author.
